# The 3D genome of *Gigaspora margarita* unveils stable chromatin and nucleolar organization and symbiont‐dependent genome dynamics

**DOI:** 10.1111/nph.71100

**Published:** 2026-03-20

**Authors:** Ken Mugambi, Jordana Oliveira, Franco Magurno, Alessandra Salvioli di Fossalunga, Mara Novero, Luisa Lanfranco, Stefano Ghignone, Gökalp Yildirir, Yan Wang, Paola Bonfante, Nicolas Corradi

**Affiliations:** ^1^ Department of Biology University of Ottawa Ottawa ON K1N 6N5 Canada; ^2^ Institute of Biology, Biotechnology and Environmental Protection University of Silesia Jagiellońska 28 Katowice 40‐032 Poland; ^3^ Department of Life Sciences and Systems Biology University of Torino 10125 Torino Italy; ^4^ Institute for Sustainable Plant Protection – Turin Unit Consiglio Nazionale delle Ricerche 10125 Torino Italy; ^5^ Department of Biological Sciences University of Toronto Scarborough Toronto ON M1C 1A4 Canada

**Keywords:** arbuscular mycorrhizal fungi, chromosome compartments, endobacterium, nucleolus, rRNA operon, transposable elements

## Abstract

- Arbuscular mycorrhizal fungi (AMF) are widespread plant symbionts that enhance nutrient acquisition and influence ecosystem productivity. Previous chromosome‐level assemblies of the model species *Rhizophagus irregularis* revealed a two‐compartment genome architecture (active A and repressed B chromatin compartments), yet its conservation across evolutionarily distant AMF lineages remains unresolved.
- Here, we present a chromosome‐scale and 3D genome assembly of *Gigaspora margarita* isolate BEG34 – the largest and most repeat‐rich AMF genome to date – alongside that of its obligate endobacterium, *Candidatus* Glomerobacter gigasporarum (*Ca*Gg), using PacBio HiFi and Hi‐C sequencing.
- The *G. margarita* genome comprises 43 chromosomes (792 Mb) organized into A/B compartments and Topologically Associating Domains, structures that are conserved across two AMF orders and remain stable irrespective of the presence of endobacteria in germinating spores. We uncover 21 divergent rDNA operons distributed across six chromosomes and show that these physically interact, suggesting conserved nucleolar organization. We also reveal that the *Ca*Gg genome is tripartite and mobilome‐rich, encoding prophages, an orphan CRISPR array, and complete pathways for many novel and essential cofactors, including heme, which may enhance host bioenergetics. We also find that the endobacterium's presence modulates transposable elements expression in *G. margarita*.
- These findings reveal conserved principles of chromatin architecture in AMF symbionts and highlight the tight molecular interplay between fungal hosts and their endosymbionts, offering new insights into genome evolution and symbiotic adaptation.

## Abstract 2

See also the Commentary on this article by Baroncelli, **251**: 2289–2291.

## Introduction

Arbuscular mycorrhizal fungi (AMF) are plant root symbionts that belong to the subphylum Glomeromycotina (Spatafora *et al*., 2016). As obligate biotrophs, AMF require a living plant host to complete their life cycle, leading them to colonize the cortical root cells and develop specialized tree‐like structures called arbuscules (Smith & Read, 2008). Within these structures, the plant supplies the fungi with lipids and sugars (Keymer *et al*., 2017; Luginbuehl *et al*., 2017), while the AMF provides the plant with essential nutrients that are limiting factors for plant growth (Bonfante, 2018), primarily phosphate, resulting in improved crop yields (Terry *et al*., 2023; MacColl & Maherali, 2025), carbon storage (Ferguson *et al*., 2025), and enhanced defense against pathogens (Pozo & Azcón‐Aguilar, 2007). AMF are always multinucleated, with individual spores carrying up to 20 000 haploid nuclei in some species (Kokkoris *et al*., 2020). Although sexual reproductive structures have not yet been observed in AMF, genome and single‐nucleus analyses have shown that these organisms carry conserved mating‐related genes (Halary *et al*., 2011, 2013; Riley & Corradi, 2013; Tisserant *et al*., 2013; Riley *et al*., 2014; Morin *et al*., 2019) and follow homokaryotic/heterokaryotic cycles (Ropars *et al*., 2016; Sperschneider *et al*., 2023), which define sexual processes in fungi (Ropars *et al*., 2012; Wallen & Perlin, 2018). Genome analyses show that AMF plant dependence is likely linked to the loss of genes involved in fatty acid production, thiamine biosynthesis, sugar utilization, and plant cell wall degradation (Tisserant *et al*., 2013; Oliveira *et al*., 2024). Their genomes are also highly enriched in transposable elements (TEs), and closely related AMF strains exhibit striking variation in gene content (Chen *et al*., 2018; Sahraei *et al*., 2022). The abundance of TEs in AMF is the main reason their genome assemblies have long been highly fragmented, thereby hampering our understanding of their genetic structure and overall genome biology.

This issue was recently addressed by combining long reads with chromatin capture (Hi‐C) datasets, which allowed the generation of chromosomal‐level assemblies for model AMF *Rhizophagus irregularis* strains (order Glomerales) (Yildirir *et al*., 2022; Sperschneider *et al*., 2023). This approach revealed that AMF chromatin separates into two compartments (A/B). The compartment A contains transcriptionally active genes, high methylation of TEs, and the most conserved core genes. By contrast, compartment B harbors transcriptionally repressed genes and is rich in genes encoding secreted proteins, candidate effectors, and TEs that are upregulated *in planta* (vs extraradical mycelium) (Kloppholz *et al*., 2011; Yildirir *et al*., 2022; Sperschneider *et al*., 2023; Teulet *et al*., 2023; Oliveira *et al*., 2024, 2025; Oliveira & Corradi, 2024). This stage‐specific upregulation suggests that root colonization leads to the relaxation of the B sub‐compartments involved in the molecular dialogues between partners of the mycorrhizal symbiosis (Yildirir *et al*., 2022; Oliveira *et al*., 2024). In support of this, transmission electron microscopy of the AMF *Gigaspora margarita* shows shifts in chromatin condensation, transitioning from a tightly packed state in spores to a looser state in intraradical hyphae during plant root colonization (Lanfranco & Bonfante, 2023).

In addition to being separated into A/B compartments, the available *R. irregularis* genomes are also organized into finer‐scale structural units known as topologically associating domains (TADs)‐like structures, within which chromatin interactions occur more frequently than with neighboring regions (Dixon *et al*., 2012; Dekker & Heard, 2015; Yildirir *et al*., 2022; Torres *et al*., 2023). In model eukaryotes, TADs are separated by ‘boundaries’ that act as insulators, limiting epigenetic interactions between adjacent TADs, and are often enriched for protein‐coding genes and depleted of DNA repeats (Dixon *et al*., 2012; Kurbidaeva *et al*., 2025). By contrast, in fungal species, repeats were shown to dominate TAD boundaries (Winter *et al*., 2018), highlighting their functional divergence and potentially higher malleability. Hi‐C‐based identification of A/B compartments in AMF remains confined to the model AMF species *R. irregularis*, and independent support for their existence and conservation in other AMF lineages is lacking. Similarly, although data from model eukaryotes (Hansen *et al*., 2018; Zhang *et al*., 2023; Li *et al*., 2024) supports the hypothesis that the AMF A/B sub‐compartments may change their 3D chromatin structure in response to host and environmental factors, direct evidence based on chromatin analyses that such changes occur in AMF has not yet been provided.

Here, we aim to fill these knowledge gaps by sequencing the genome of *G. margarita* isolate BEG34 (order Diversisporales) using PacBio HiFi and Hi‐C data. This species contains the largest and most repeat‐rich AMF genome known to date (Venice *et al*., 2020). It also carries beneficial obligate *Burkholderia*‐like endobacteria (*Candidatus* Glomeribacter gigasporarum; *Ca*Gg) within its cytoplasm, which have been shown to impact fungal biology (Salvioli *et al*., 2016; Bonfante & Desirò, 2017; Turina *et al*., 2018). The presence of *Ca*Gg in *G. margarita* BEG34 spores (+*Ca*Gg) and the availability of a cured line of the same strain (−*Ca*Gg), along with its large and repeated genome and phylogenetic placement in the AMF order Diversisporales, collectively position *G. margarita* as an ideal species to elucidate diversity and conservation of AMF chromosome biology and the plasticity of A/B compartments and TADs.

## Materials and Methods

### Sample preparation for HiFi sequencing

Spores of *Gigaspora margarita* Becker and Hall (BEG 34, deposited at the European Bank of Glomeromycota) containing (+*Ca*Gg) or lacking (−*Ca*Gg) the obligate endobacterium *Candidatus* Glomeribacter gigasporarum were used in this study. The −*Ca*Gg spores were obtained from +*Ca*Gg spores as described in (Lumini *et al*., 2007). The +*Ca*Gg and −*Ca*Gg spores were isolated from clover trap plants by wet sieving (Gerdemann & Nicolson, 1963) and collected individually under a dissecting microscope. Only the healthiest spores were selected and pooled into groups of 100, then surface‐sterilized with a solution of Chloramine T (3% w/v) and streptomycin sulfate (0.03% w/v) in sterile distilled water. A batch of +*Ca*Gg spores was immediately frozen in liquid nitrogen, while the remaining batches (+*Ca*Gg and −*Ca*Gg) were incubated to allow germination.

### DNA extraction and purification

DNA was extracted from pools of 100 sterilized and frozen +*Ca*Gg spores using the DNeasy Plant Pro Kit (Qiagen). The spores were ground in the extraction buffer using a TissueLyser homogenizer (2 min at 24 Hz, repeated twice), and DNA extraction was performed according to the manufacturer's instructions, except that the PS solution was omitted and the samples were not heated. DNA from two independent pools of 100 spores each was combined, and a cleanup step was performed with the DNeasy PowerClean Pro Kit (Qiagen) according to the manufacturer's instructions.

### Sample preparation and Hi‐C sequencing

For Hi‐C analysis, 2900 +*Ca*Gg and 2900 −*Ca*Gg sterilized spores were placed in multiwell plates containing 1 ml of sterile distilled water and incubated in the dark at 30°C for 7 d to allow germination. Germinated spores and pre‐symbiotic mycelium were fixed at room temperature under gentle shaking for 20 min in 1% (v/v) formaldehyde in 0.01 M PBS (pH 7.2), followed by an additional 15 min incubation in 0.125 M glycine in 1% formaldehyde in 0.01 M PBS (pH 7.2). Spores and mycelium were then frozen in liquid nitrogen and ground with sterile micropestles to a fine powder. Samples were stored at −80°C until library preparation was performed.

Hi‐C data was processed using the Proximo Hi‐C Kit (Fungal) from Phase Genomics (Seattle, WA, USA), employing four restriction enzymes: *Dpn*II, *Mse*I, *Dde*I, and *Hin*FI. The Hi‐C libraries were sequenced on the Illumina NovaSeq X PE150 platform, generating 194 and 202 million reads for the −*Ca*Gg and +*Ca*Gg samples, respectively (Supporting Information Table S1).

### Genome assembly and Hi‐C scaffolding

A total of 9.88 million PacBio HiFi reads were obtained on the Revio Platform at Mount Sinai Hospital (Toronto) and assembled into contigs using Hifiasm v.0.16.1 with the parameters ‐l0 (no purging) and ‐‐h1 and ‐‐h2 for Hi‐C data integration (Cheng *et al*., 2022). The raw assembly was queried against the NCBI nr database using Diamond blastx (v.0.9.14.115; perc_identity 75, −evalue 1e‐5) to detect and remove contaminants, and identify mitochondria and *Ca*Gg contigs, which were annotated using MitoHifi v.3.2.3 (Uliano‐Silva *et al*., 2023) and Bakta v.1.8.2 (Schwengers *et al*., 2021), respectively.

The remaining contigs were scaffolded using the Hi‐C reads, which were first processed using the Arima Genomics pipeline (Arima Genomics, 2019). The resulting BAM file alignments were used as input to the YaHS scaffolding tool (Zhou *et al*., 2023). The assembled scaffolds were manually curated using PretextView (Harry, 2020) to generate chromosome‐scale scaffolds and identify the unplaced contigs. A Python script was used to identify telomeres (https://github.com/JanaSperschneider/FindTelomeres). The final chromosome‐scale scaffolds were visualized using the genome‐wide Hi‐C heatmap generated by Juicebox Assembly Tools v.1.11.08 (Durand *et al*., 2016) and through a chromosome ideogram layout using RIdeogram (Hao *et al*., 2020).

### Repeat masking and transposable elements analysis

The assembled genome was submitted to RepeatModeler2 (Flynn *et al*., 2020) to generate a consensus library of repetitive sequences. To better classify repeats and address the high number of unclassified sequences (‘Unknown’), we curated the consensus library using TEtrimmer (Qian *et al*., 2025) and manual curation, as has been previously done for other AMF species (Oliveira & Corradi, 2024; Oliveira *et al*., 2025). Using this method, sequences known to be expanded genes were removed. The genome masking and TEs annotation were performed on the final library, which contains a curated consensus of the assembled genome using RepeatMasker (Smit *et al*., 1996).

RNA‐seq data from intraradical mycelium (IRM) with and without the endobacterium, from colonizing *Lotus japonicus* roots, and from extraradical mycelium (ERM) of *G. margarita* were obtained from a previously published study (Venice *et al*., 2021) (BioProject PRJNA267628), which provides detailed experimental procedures. These datasets were used to assess TE differential expression. The reads were first aligned to the reference genome assembled in this study using HISAT2. TE expression was then evaluated using TEtranscripts (Jin *et al*., 2015), applied to the aligned reads, guided by the gene and TE annotations carried out in this study.

### Genome annotation

Gene prediction was performed on a soft‐masked genome assembly using the Funannotate pipeline v.1.8.15 (Palmer & Stajich, 2020). First, the command ‘funannotate train’ was executed to train *ab initio* gene predictors with previously published RNA‐seq data (PRJNA751155) (Venice *et al*., 2021). Next, the command ‘funannotate predict’ was run with the parameters ‐‐optimize_augustus and ‐‐ploidy 1. Multiple sources of evidence were utilized as input during the prediction of protein‐coding sequences: (1) transcript assemblies (‐‐transcript_evidence) and alignments (‐‐rna_bam); (2) gene models generated by PASA (‐‐pasa_gff); and (3) protein sequences from UniProtKB and the *G. margarita* proteome. The quality of the annotated genome was assessed using Benchmarking Universal Single‐Copy Orthologs v.5.2.2 (BUSCO) (Simão *et al*., 2015). SignalP v.6.0 (Teufel *et al*., 2022) was employed to predict secreted proteins with parameters ‐‐organism eukarya and ‐‐mode slow. EffectorP v.3.0 (Sperschneider & Dodds, 2022) was utilized to predict effector genes, and carbohydrate‐active enzymes (CAZymes) were annotated using dbCAN (Huang *et al*., 2018; Zheng *et al*., 2023). To identify B12‐dependent enzymes in *G. margarita*, homologs previously identified in *R. irregularis* (Orłowska *et al*., 2021) were used as BLASTP queries.

### Phylogenetic analyses

To infer the phylogenetic relationships among SSU‐ITS‐LSU rDNA paralogs found in *G. margarita* and rDNA copies from other *Gigaspora* species, a dataset was created including sequences of *Dentiscutata savannicola* and *Fuscutata heterogama* as outgroups. Members of *Paradentiscutata* and *Intraornatospora*, the closest genera to *Gigaspora*, were not chosen as outgroups because they only have partial LSU sequences. Among the 21 rDNA operons of *G. margarita*, 19 were used to overlap the partial SSU‐ITS‐LSU region used for phylogenetic inference. Overall, the dataset comprised 96 sequences representing eight *Gigaspora* species and 10 outgroup sequences. The dataset was aligned with the online version of MAFFT v.7 (Katoh & Standley, 2013), using the E‐INS‐i iterative refinement method, and edited in MEGA v.5.2.2. by manual trimming of overarching and misaligned ends. Maximum likelihood phylogenetic inference was carried out in RAxML‐NG via CIPRES Science Gateway 3.1 (Miller *et al*., 2011) and in IQ‐TREE v.2.2.5 (Minh *et al*., 2020), with 1000 bootstrap, 1000 ultrafast bootstrap replicates and 1000 SH‐aLRT tests, respectively. Additionally, a Bayesian analysis was performed in MrBayes v.3.2.6 with 40 million generations and a stop rule at split frequency SD = 0.01. All analyses were conducted with partitions and nucleotide substitution models as previously described (Magurno *et al*., 2024). Notably, we found that most ingroup branches were either unsupported or poorly supported, particularly with respect to bootstrap and posterior probability values. In the Bayesian analysis, after 40 million generations, the average SD of split frequencies did not approach 0.01 but instead fluctuated *c*. 0.055, suggesting a potential lack of convergence in some bipartitions.

To determine the placement of *G. margarita* in the AMF phylogeny, phylogenomic analysis was performed using the ‘fungi_odb10’ dataset from the BUSCO v.4.0 (Simão *et al*., 2015). Profile‐Hidden‐Markov‐Models corresponding to these markers were used to identify homologous sequences in *G. margarita* and 50 additional fungal genomes, using HMMER3 v.3.1b2 as implemented in the PHYling pipeline (https://github.com/stajichlab/PHYling). A total of 702 conserved, single‐copy orthologous proteins were identified, aligned, and concatenated for downstream phylogenetic inference. The phylogenetic tree was reconstructed using a maximum likelihood approach, with the best‐fit substitution model selected for each gene partition in IQ‐TREE v.1.6.12. The final analysis was based on 604 857 aligned sites, with 588 555 distinct patterns contributing to the tree topology.

### Methylation and RNA‐seq analyses

PacBio HiFi reads with kinetic data were processed using Jasmine v.3.0.0 (jasmine, 2023). The output, which identified 5mC sites, was saved as ML and MM tags in the unaligned BAM file. The reads were then aligned to the *G. margarita* genome using pbmm2 1.10.0 (pbmm2, 2017) with the ‐preset HiFi parameter. PacBio CpG tools (pb‐CpG‐tools, 2022) were then used to calculate CpG methylation frequency, using the options pileup_calling_model v.1, tflite model, and ‐min‐mapq 0. The resulting CpG locations were then intersected with genome‐wide compartments, genes, and repeat regions using bedtools v.2.30.0 (Quinlan & Hall, 2010) to determine median methylation frequencies within 50‐kb windows.

For gene expression analysis, RNA‐seq datasets from *G. margarita* germinating spores, ERM, and intraradical mycelium colonizing *L. japonicus* roots were mapped to the transcriptome using Salmon v.1.3.0 (Patro *et al*., 2017). The transcriptome was first indexed using the salmon index module with the ‐keepDuplicates option, and reads were quantified with salmon quant, specifying the ‐validateMappings parameter. The Transcripts Per Million values from the salmon output were log‐transformed and compared between A/B compartments across the three RNA‐seq datasets.

### Compartment and TADs analysis

HiC‐Pro v.2.11.1 (Servant *et al*., 2015) and HicExplorer were used to process, analyze, and visualize Hi‐C data. First, HiC‐Pro was used to map and filter +*Ca*Gg and −*Ca*Gg Hi‐C reads, retaining only alignments with a MAPQ score greater than 10. Contact matrices were then generated at multiple resolutions, ranging from 20 kb to 100 kb in 10 kb increments. The resulting contact matrices were converted to HDF5 (.h5) format using the hicConvertFormat module in HicExplorer. The eigenvector decomposition implemented in the hicPCA command of HicExplorer was used to call A/B compartments from the contact maps. The first eigenvector (PC1) corresponded to the A and B compartments, and the direction of the PC1 values (positive or negative) was used to determine the compartment identity. Specifically, the contact matrices were manually examined per chromosomal scaffold, and regions with identical positive values were labeled ‘1’, while those with negative values were labeled ‘2’. Finally, to assign A/B compartments, the label associated with higher average RNA‐seq gene expression and gene density was assigned to compartment A, while the opposite label was assigned to compartment B.

To assess whether the presence of an endobacteria primes compartment switches, eigenvalues from contact matrices of *G. margarita* spores with and without endobacteria were compared. Regions that showed a change in PC1 values from positive to negative, or vice versa, were considered compartment switches. Additionally, the correlation of eigenvalues between the two conditions was calculated to determine the degree of shifts in compartmentalization.

We predicted TAD bodies and boundaries using hicFindTADs from HiCExplorer at 30 kb resolution, with the following parameters: ‐‐correctForMultipleTesting bonferroni and ‐‐thresholdComparisons 0.01. We then assessed the distribution of repetitive elements, protein‐coding genes, and methylation levels around TAD boundaries and within ±50 kb from these boundaries using BEDTools. The resulting metaplots were generated using deepTools (Ramírez *et al*., 2014). Next, we compared gene expression (TPMs) patterns of boundary‐associated genes and non‐boundary genes across three conditions: germinating spores, ERM, and *in planta* (lotus). Additionally, to explore whether genes within the same TAD show coordinated expression, we calculated the coefficient of variation for TAD and non‐TAD regions.

### *Ca*Gg genomic analyses

To confirm the number and circularity of the *Ca*Gg contigs, we reassembled HiFi reads using Flye v.2.9.6 (Kolmogorov *et al*., 2019) and Unicycler v.0.5.1 (Wick *et al*., 2017) genome assemblers. This process resulted in three *Ca*Gg contigs, all of which were verified as circular. One contig, containing core bacterial genes, was identified as the chromosome, while two smaller contigs containing plasmid‐associated genes were classified as plasmids. The *Ca*Gg circular genome map was generated using GenoVi v.0.2.16 (Cumsille *et al*., 2023). To identify Type IV Secretion System (T4SS) and Mobilization enzymes (MOB), SecReT4.0 (https://bioinfo‐mml.sjtu.edu.cn/SecReT4/) and MOBscan (https://castillo.dicom.unican.es/mobscan/) were employed. Prophage regions were located using Phaster (https://phaster.ca/) with default settings. Phaster categorizes predicted prophage sequences as ‘intact’ (score ≥ 90), ‘Questionable’ (score 70–90), and ‘Incomplete’ (score < 70), based on size, the presence of phage‐related genes, and similarity to known phages. Predicted protein sequences were assigned KEGG Orthologs (KOs) and mapped to KEGG pathways with KofamKOALA (www.genome.jp/tools/kofamkoala/). The type II TA system was predicted using TAfinder 2.0 (https://bioinfo‐mml.sjtu.edu.cn/TADB3/TAfinder.php) (Guan *et al*., 2024). CRISPRCasFinder v.4.2.20, implemented in Prokesee, was used to identify CRISPR arrays and cas genes.

## Results

### Chromosome‐level assembly, annotation, and phylogenomics of *G. margarita*

**Fig. 1:**
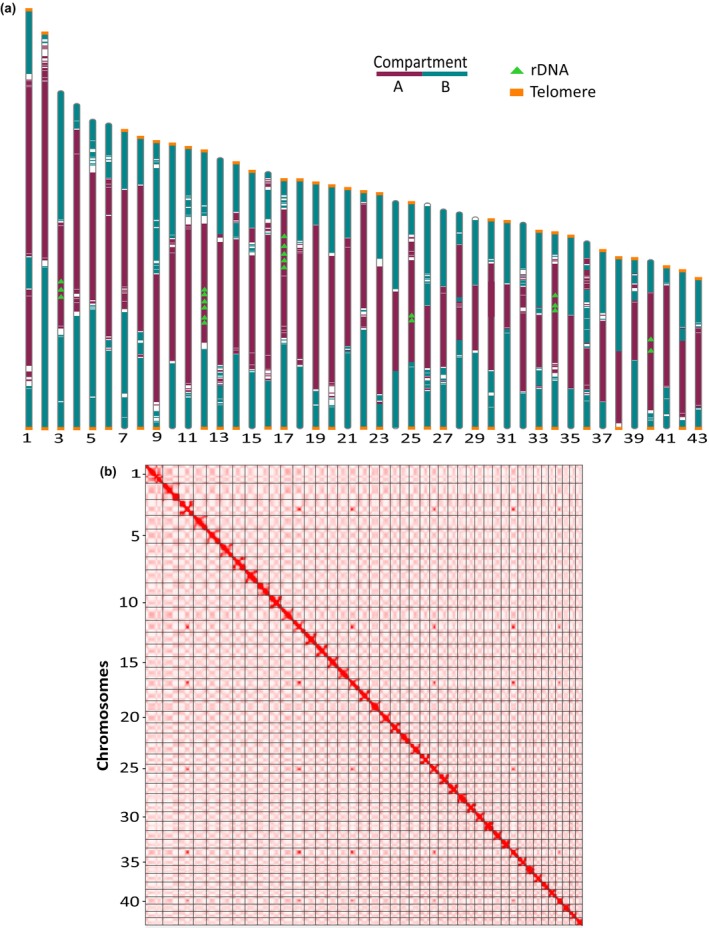
*Gigaspora margarita* chromosomes and genome content. (a) Karyoplots of 43 chromosomes, with each rDNA repeat (18S‐5.8S‐28S) shown as a lime‐green triangle, A/B compartments as violet‐red and turquoise bars, respectively, a white bar indicating unassigned compartment identity within the ideograms, and rectangular orange bars denoting telomers. (b) Genome‐wide Hi‐C contact map of *G. margarita*. The black squares represent chromosomes sorted by size. The color intensity corresponds to interaction frequencies between loci, with darker red indicating high contact probability and white signifying low or no interactions.

We performed PacBio and Hi‐C sequencing of genomic DNA extracted from *G. margarita* +*Ca*Gg spores. Using Hifiasm with Hi‐C integration mode (Cheng *et al*., 2021), we assembled the HiFi long reads into draft contigs, which were then scaffolded using Hi‐C data (Cheng *et al*., 2021; Sperschneider *et al*., 2023). For *G. margarita*, this approach generated 43 chromosome‐level scaffolds, with a genome coverage of 82X, a size of 792.14 Mb and an N_50_ of 18.89 Mb (Table 1; Fig. 1a,b). Of these, 20 chromosomes have telomeres at both ends, 20 have telomeres at only one end, and three lack telomeres entirely. Only 23 contigs (0.718 Mb) could not be assigned to any chromosome. This assembly represents a significant improvement over the previously available datasets (Venice *et al*., 2020) in assembly size (792.14 Mb vs 773.10 Mb), fragmentation (43 vs 6490 scaffolds), and contiguity (N_50_ of 18.89 Mb vs 326.79 kb).

**Table 1:** Summary statistics for the genome assembly of *Gigaspora margarita*.

Feature	*Gigaspora margarita*
Genome size (Mbp)	792.14
No. of scaffolds	43
No. of genes	30 211
No. of rDNA repeats (18S‐5.8S‐28S)	21
Repeat content (%) (after curation)	78.28
BUSCO completeness (%)	95.4
GC (%)	27.77

Genome annotation identified 30 211 protein‐coding genes, resulting in a BUSCO completeness of 95.4% (Table 1). Of these, 2438 (8.1%) were annotated as putative secreted proteins and effectors, numbers higher than those reported in *R. irregularis* (Table S2). We confirm that *G. margarita* lacks the hallmark ‘Missing Glomeromycota Core Genes (MGCGs)’ (Tang *et al*., 2016) (Table S3) and has a reduced set of carbohydrate‐active enzymes (CAZymes). However, compared with *R. irregularis*, it shows a significant expansion of CAZyme families involved in chitin metabolism (GH18, GT1, GT2, AA7, and CE4) (Venice *et al*., 2020; Rosling *et al*., 2024) (Table S4). The annotation also uncovered cobalamin‐dependent enzymes in *G. margarita* (Table S5), supporting the hypothesis that the fungus uses cobalamin supplied by *Ca*Gg (Ghignone *et al*., 2012). The chromosome‐level assembly confirms that all AMF with sequenced genomes carry all known meiosis‐specific genes. It further supports that, as opposed to most AMF with sequenced genomes (Oliveira *et al*., 2024), including representatives of early‐branching lineages (Malar *et al*., 2021, 2022), the genes composing the putative AMF mating‐type (Ropars *et al*., 2016; Sperschneider *et al*., 2023), namely a choline *trans*porter, two homeodomain proteins (HD1‐2), and a phosphate glycerate mutase, are not adjacent within members of the Gigasporaceae (Morin *et al*., 2019). Alongside the nuclear genome, we recovered the complete mitochondrial genome as a single contig. The genome size is 96 986 bp and encodes core mitochondrial genes, including 19 protein‐coding genes, 2 *trans*‐spliced rRNA genes, and 24 tRNA genes, consistent with previous findings for the *G. margarita* mitochondrion (Pelin *et al*., 2012).

We took advantage of this new chromosome‐level dataset to determine the evolutionary placement of *G. margarita* relative to other AMF species via phylogenomic analyses (Fig. S1). These analyses support the sister relationship between Glomeromycotina and Mortierellomycotina within the Mucoromycota phylum (Spatafora *et al*., 2016; Wijayawardene *et al*., 2022). It also backs the close evolutionary relationship between Glomerales and Diversisporales, the paraphyletic placement of *Entrophospora* species (Beaudet *et al*., 2018; Malar *et al*., 2021, 2022; Błaszkowski *et al*., 2022; Rosling *et al*., 2024), as well as the early‐branching of Paraglomerales and Archeosporales within the AMF phylogeny. Notably, in support of phylogenetic analyses based on ribosomal genes (Redecker *et al*., 2013) and available genome datasets (Malar *et al*., 2022), our findings highlight Paraglomerales as representing the earliest AMF phylogenetic node. However, we were unable to fully reject an alternative placement of *P. occultum* with members of the Archaeosporales.

### The divergent rDNA repeats are physically linked in *G. margarita*

The chromosome‐level genome annotation also confirms that, unlike most eukaryotes, AMF carry few copies of highly divergent ribosomal DNA (rDNA) operons within their genomes. The *G. margarita* genome has 21 rDNA copies, approximately twice as many as *R. irregularis* strains (Yildirir *et al*., 2022), and these vary in copy number and sequence both within and across chromosomes 3, 12, 17, 25, 34 and 40 (Fig. 1a,b; Table S6). The high rDNA sequence divergence within members of Glomerales was reported to cause significant taxonomic challenges, as rDNA paralogs can sometimes cluster across species boundaries (Maeda *et al*., 2018; Corradi *et al*., 2025). We find that this issue is exacerbated in *Gigaspora* species, further building concerns about the utility of these genes alone for AMF taxonomy (Maeda *et al*., 2018; Corradi *et al*., 2025; Stefani *et al*., 2025). For instance, the rDNA paralogs in the *G. margarita* genome scatter across three clades and are shared with sequences from *G. decipiens*, *G. albida*, and *G. gigantea*. Almost all *Gigaspora* species show similar paraphyletic clustering based on rDNA paralogy rather than speciation (Fig. S2).

**Fig. 2:**
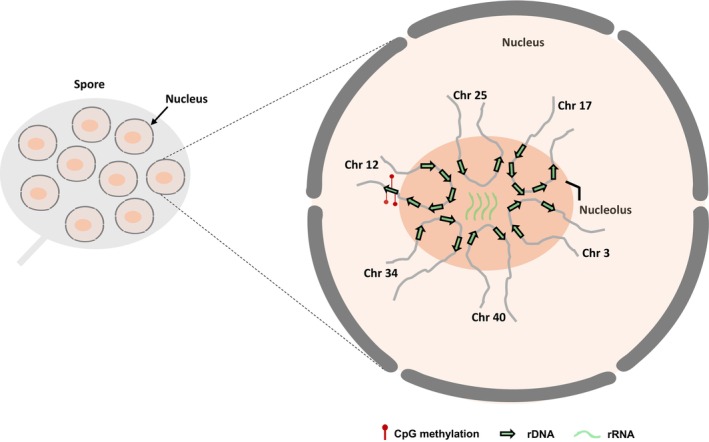
A working model of the rDNA 3D organization within the AMF nucleolus. Based on current Hi‐C data and knowledge of rRNA transcription in model eukaryotes. Transcriptionally active rDNA repeats from different scaffolds hypothetically interact physically in the nucleolus. The methylated rDNA copy sits outside the nucleolus. Red lollipops indicate CpG methylation; arrows (black outline, lime‐green fill) depict rDNA repeats; lime‐green wavy lines represent rRNA.

In addition to enabling chromosome‐level assemblies, the Hi‐C data also revealed strong physical interactions among rDNA copy regions on chromosomes 3, 12, 17, 25, 34, and 40, as evidenced by visually bright signals off the diagonal line in Hi‐C maps (Fig. 1b; Table S6). In model eukaryotes, active rDNA copies physically cluster in the nucleolus to allow rDNA biogenesis, while inactive copies are found at its edges or outside (Boisvert *et al*., 2007; Pederson, 2011; Schöfer & Weipoltshammer, 2018). A very similar pattern is seen in *G. margarita*: most rDNA units are hypomethylated (i.e. actively transcribed), with only one unit, located on chromosome 12, highly methylated, and thus possibly transcriptionally silenced. Notably, investigations of Hi‐C data from *R. irregularis* strains uncovered similar rDNA interactions between regions on chromosomes 9, 18, 23, and 28 (Fig. S3). Taken together, our results indicate that AMF divergent rDNAs follow a cellular mechanism found in model eukaryotes, in which active rDNA units are physically close to each other within the nucleus, presumably residing within the nucleolus, while inactive units are positioned at the periphery of the nucleolus or outside it (Fig. 2).

### Endobacteria‐driven regulation of transposable elements in *G. margarita*

**Fig. 3:**
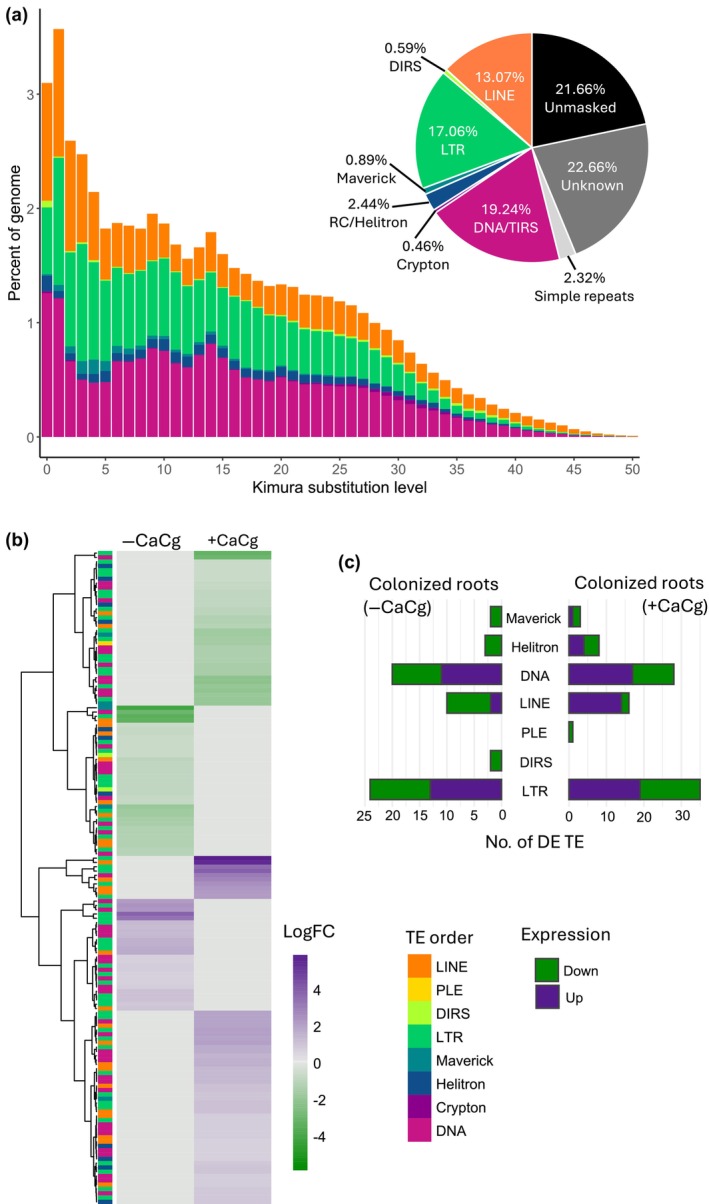
Composition and activity of transposable elements in *Gigaspora margarita* genome. (a) Transposable element distribution and repeat landscape. The pie chart depicts the relative abundance of each type of sequence in the *G. margarita* genome. ‘Unmasked’ denotes the portion of the genome that is non‐repetitive. The histogram represents the repeat landscape grouped by Kimura divergence levels (*x*‐axis) in relation to the genome (*y*‐axis). (b) Heatmap for differentially expressed TE families during *Lotus japonicus* root colonization, comparing samples lacking the *Ca*Cg endobacterium (−*Ca*Cg) with *Ca*Cg‐containing samples (+*Ca*Cg) in relation to spores (control). Rows represent TE families, annotated by TE order and clustered by expression profile. (c) Number of differentially expressed TE families by order and direction of regulation (up‐ or downregulated) in colonized roots, shown separately for samples without and with *Ca*Cg.

The genome of *G. margarita* is known to be highly repetitive, but the true extent and diversity of these repeats were likely obscured by the fragmented nature of available genome datasets (Muszewska *et al*., 2019) and a lack of curated analysis of such repeats (Oliveira *et al*., 2025). Curating our chromosome‐level assembly revealed that 78.34% of the *G. margarita* genome consists of repeats, of which 56.1% belong to known TE families. Among these, 19.2% correspond to DNA/TIRS (Terminal Inverted Repeat) elements (i.e. DNA transposons), while 17.1% and 13.1% are Long Terminal Repeat (LTR) and Long Interspersed Nuclear Element (LINE) retrotransposons, respectively. Overall, 22.7% of the repeats remain unknown and cannot be classified after manual curation (Fig. 3), which compares to 41% in previous work (Venice *et al*., 2020). These unknown repeats may represent expanded gene families commonly found in AMF (Dallaire *et al*., 2021; Oliveira *et al*., 2025), as well as TE families that have not yet been formally characterized in model species, including fungi. We assessed the expansion and degeneration of TEs in *G. margarita* by constructing their repeat landscapes based on Kimura substitution calculations (Fig. 3), where lower Kimura substitution rates indicate recent TE insertions, while higher rates suggest older insertions (Dallaire *et al*., 2021). Our analyses build on previous findings showing that TEs in Diversisporales are enriched in recent and active expansions (Oliveira *et al*., 2025), with DNA/TIR elements, LINEs, and LTRs being the primary contributors to recent TE bursts in *G. margarita*.

In AMF, including *G. margarita*, TEs are preferentially located close to promoters and genes involved in molecular communication with the host (e.g. effectors) (Oliveira *et al*., 2025), which indicates their potential role in regulating gene expression and maintaining successful mycorrhizal relationships. Reports of significant TE upregulation in the model AMF *R. irregularis* following root colonization supported this view (Oliveira & Corradi, 2024). A more comprehensive annotation of TEs allowed a detailed analysis of the differential regulation of fungal TEs in *L. japonicus* roots colonized by *G. margarita*, with and without endobacteria. Remarkably, the presence of the bacterial endosymbiont alters the regulation of specific TE families during root colonization (Fig. 3b,c). Among these, some members of the Helitron, Maverick, and LINE families were specifically upregulated only in the presence of *Ca*Cg, while members of the *Penelope*‐like elements (PLE) family were upregulated exclusively in the absence of *Ca*Cg. Furthermore, *Dictyostelium* intermediate repeat sequence (DIRS) families were downregulated in the absence of *Ca*Cg (Fig. 3c).

### Identification of A/B compartments and topologically associating domains in *G. margarita*

**Fig. 4:**
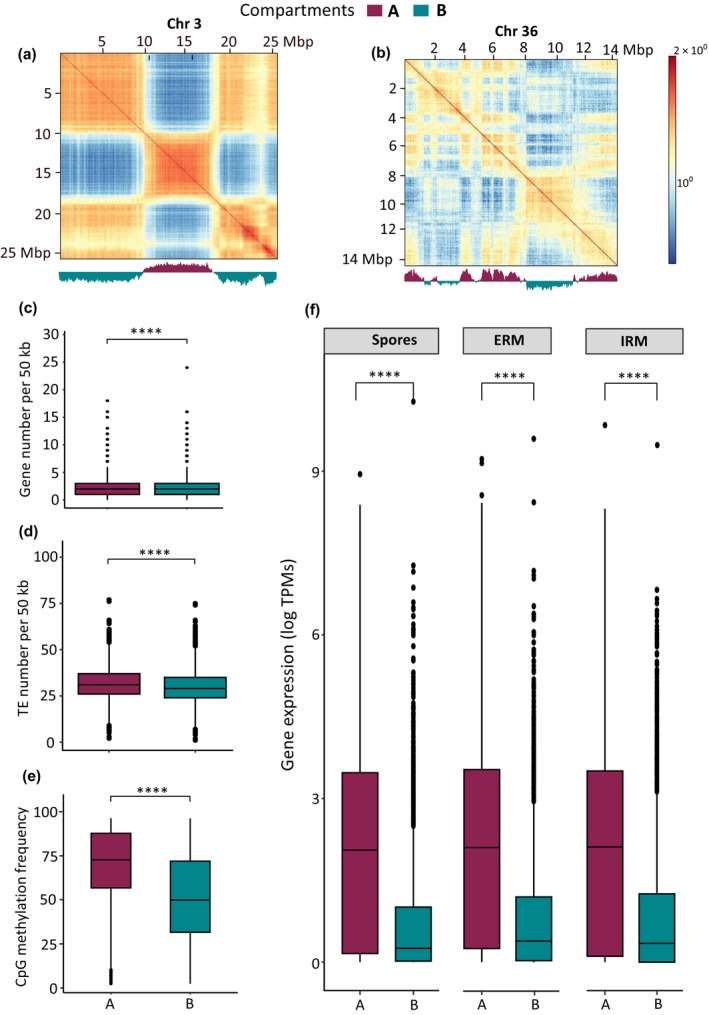
Genome compartmentalization in *Gigaspora margarita*. Examples of Hi‐C contact maps showing A/B compartments in *G. margarita* chromosomes (a) 3 and (b) 36. The bottom track shows the first eigenvector values, identifying the compartment interaction at 50‐kb resolution. Regions that interact more frequently are visualized as brighter squares on the contact map. Gene/repeat densities, methylation frequency, and gene expression in compartments A and B of *G. margarita*. Boxplots showing (c) Genes (Genes per 50 kb), (d) repeat densities (Repeats per 50 kb), (e) CpG methylation frequency, and (f) gene expression levels (logTPM +1) in three conditions: Spores, extraradical mycelium (ERM), and intraradical mycelium (IRM) (*Lotus japonicus*) in A and B compartments. Boxes show the first quartile (25%), the middle black line (50%), and the third quartile (75%). The whiskers extend to 1.5× the box length, representing the outliers as dots. Asterisks above the boxplots indicate significant differences between the A/B compartments (*P* < 0.05, Wilcoxon rank‐sum test).

Hi‐C data also revealed that the *G. margarita* genome exhibits a checkered pattern delineating two A/B compartments (Figs 4a,b, S4), as reported in all *R. irregularis* strains with available Hi‐C data (Yildirir *et al*., 2022; Sperschneider *et al*., 2023). Some chromosomes carry large blocks of A or B compartments (Fig. 4a), while others harbor a finely interleaved, plaid‐like pattern of A/B compartmentalization (Fig. 4b).

The *G. margarita* A compartment has significantly higher gene density and average gene expression compared to the B compartment (Fig. 4c), and contains most core genes and all rDNA operons, while the B compartment is significantly enriched for secreted proteins and candidate effector genes (Table S7). Although both compartments have similar TE densities (Fig. 4d), the TEs in the A compartment are significantly more methylated (Fig. 4e). Like *R. irregularis* homokaryons (Dallaire *et al*., 2021; Yildirir *et al*., 2022), *G. margarita* shows a strong bimodal distribution of methylation levels, with a larger percentage of CpG sites either highly methylated (> 8 = 58.8%) or weakly methylated (< 2 = 38.6%). Notably, A/B compartments remain remarkably stable between the +*Ca*Cg and ‐*Ca*Cg conditions – that is, no significant change in checkered patterns was observed both within and among chromosomes. In *G. margarita*, the A compartments are preferentially located within chromosome cores (Fig. 1), while B compartments are generally found at the chromosome ends. This clear distinction in A/B localization within chromosomes is not present in *R. irregularis* isolates (Yildirir *et al*., 2022).

**Fig. 5:**
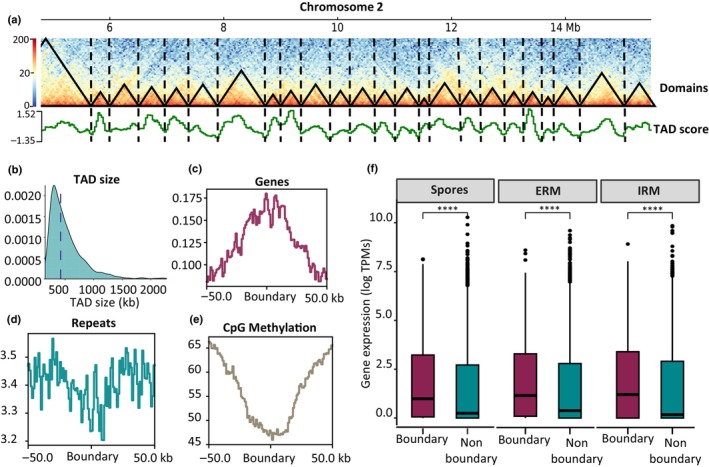
Topologically associating domains (TADs)‐like structures in the *Gigaspora margarita* genome. (a) A representative section of chromosome 2 showing examples of TADs as black triangles. The bottom green line corresponds to the insulation score (TAD score), and the vertical black dashed lines highlight the predicted TAD. (b) Density plot showing TAD size distribution. The vertical dashed line represents the mean value. (c–e) Line plots of (c) genes, (d) repeats, and (e) DNA methylation (CpG) at domain boundaries and ± 50 kb from boundaries. (f) Gene expression (log TPMs +1) in boundary and non‐boundary regions across three conditions: Spores, extraradical mycelium (ERM), and intraradical mycelium (IRM) (*Lotus japonicus*), respectively. Boxes show the first quartile (25%), the middle black line (50%), and the third quartile (75%). The whiskers extend to 1.5× the box length, representing the outliers as dots. Asterisks above the boxplots indicate significant differences between the boundary and non‐boundary regions (*P* < 0.05, Wilcoxon rank‐sum test).

Hi‐C data analysis also revealed 1407 TAD‐like structures in *G. margarita* (Fig. 5a). The TADs cover 92.16% of the genome, with a median size of 420 kb (Fig. 5b), in line with reports from other eukaryotes, including non‐AMF fungi (Dekker & Heard, 2015; Li *et al*., 2022; Torres *et al*., 2023).

We identified TAD boundaries based on low insulation scores and, surprisingly, found that they are gene‐rich and depleted of repeats (Fig. 5c,d). The gene enrichment at boundary regions is linked to low methylation levels (Fig. 5e) and, accordingly, boundary‐associated genes have significantly higher expression levels across multiple life stages, including germinating spores, extraradical, and intraradical mycelium (*L. japonicus*), compared to non‐TAD boundaries (Fig. 5f). Moreover, genes within the same TAD body are co‐expressed across life stages (Fig. S5), indicating that TAD organization facilitates transcriptional coordination in these prominent symbionts.

### The tripartite *Ca*Gg endosymbiotic genome supports genomic plasticity and reveals novel pathways for enhanced stress defense and cofactor biosynthesis

**Fig. 6:**
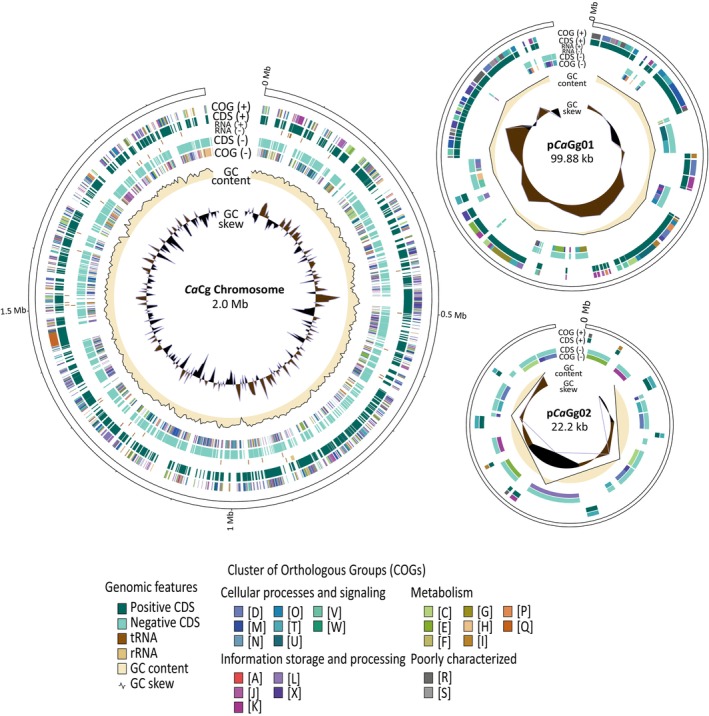
Circular genome map of *Ca*Gg. From outside to inside: + strand clusters of orthologous genes (COGs); coding sequences (CDS) on the + strand; rRNA and tRNA on the + strand; CDSs, rRNA, and tRNA on the – strand; COGs on the – strand; GC content; GC skew (+GC skew is shown as outward peaks; − as inward peaks). The COG category letters represent: J, Translation, ribosomal structure and biogenesis; A, RNA processing and modification; K, Transcription; L, Replication, recombination and repair; B, Chromatin structure and dynamics; D, Cell cycle control, cell division, chromosome partitioning; Y, Nuclear structure; V, Defense mechanisms; T, Signal transduction mechanisms; M, Cell wall/membrane/envelope biogenesis; N, Cell motility; Z, Cytoskeleton; W, Extracellular structures; U, Intracellular trafficking, secretion, and vesicular transport; O, Posttranslational modification, protein turnover, chaperones; X, Mobilome (prophages, transposons); C, Energy production and conversion; G, Carbohydrate transport and metabolism; E, Amino acid transport and metabolism; F, Nucleotide transport and metabolism; H, Coenzyme transport and metabolism; I, Lipid transport and metabolism; P, Inorganic ion transport and metabolism; Q, Secondary metabolites biosynthesis, transport and catabolism; R, General function prediction only; S, Function unknown.

Alongside the AMF genome, we obtained the complete genome of the *Ca*Gg endosymbiont, comprising a single circular chromosome of 1998 997 bp and two circular plasmids, referred to herein as p*Ca*Gg01 (99 883 bp) and p*Ca*Gg02 (22 198 bp), with a read depth double that of the chromosome (Table 2; Fig. 6). The endobacterial circular chromosome and plasmid assemblies contain the expected Origin of Replication (ORI) motifs and were all corroborated by uniform read coverage, as well as Hi‐C contact mapping (Fig. S6) and independent genome assemblers, including Flye (Kolmogorov *et al*., 2019) and Unicycler (Wick *et al*., 2017).

**Table 2:** Genome statistics and mobile genetic elements (MGEs) of the *Ca*Gg chromosome and associated plasmids p*Ca*Gg01 and p*Ca*Gg02.

Feature	*Ca*Gg chromosome	p*Ca*Gg01	p*Ca*Gg02
Size (bp)	1998 997	99 883	22 198
GC (%)	54.80	53.06	47.04
CDSs	2292	130	30
Coverage	27	55	56
MGEs			
Integration/Excision (IE)	102	2	0
Replication/Recombination/Repair (RRR)	21	0	0
Phages (P)	22	0	0
Stability/Transfer/Defense (STD)	47	5	0
Transfer (T)	15	8	0

This new assembly recovers substantially more coding capacity and non‐coding features than the previously fragmented version did. Specifically, our re‐sequenced genome assembly, totaling 2121 078 bp, is 20% larger than the previous fragmented assemblies obtained with pyrosequencing and fosmid libraries, which reported 3 ORIs in 4 genomes at 15× coverage across 125 contigs (Ghignone *et al*., 2012). It is also complete, fully resolving previously undetected plasmid sequences. The *Ca*Gg chromosome contains 2292 protein‐coding genes, 44 tRNAs, 21 non‐coding RNAs, one transfer‐messenger RNA, and a single complete rRNA operon. The plasmid p*Ca*Gg01 has 130 coding genes and one ncRNA, while p*Ca*Gg02 has 30 coding genes. A cluster of Orthologous Genes (COGs) analysis reveals that the *Ca*Gg chromosome contains a high proportion of genes associated with mobilome (X), while p*Ca*Gg01 is dominated by genes classified as defense (V), and p*Ca*Gg02 mainly contains genes involved in signal transduction (T) (Fig. 6).

The metabolic reconstruction of the *Ca*Gg chromosome confirms previously reported core metabolic modules, including the absence of phosphofructokinase (*pfk*), fatty acid degradation genes, reduced amino acid biosynthesis enzymes, and a complete cobalamin (B12) biosynthesis pathway. The endosymbiont also encodes multiple membrane transport systems, including ABC transporters and members of the major facilitator superfamily (MFS), as well as type II, III, and IV secretion systems, a complete general secretory pathway, and a twin‐arginine translocation pathway (Ghignone *et al*., 2012).

Our improved assembly also uncovered novel and biologically relevant metabolic capabilities of *CaGg*. These include complete pathways for coenzyme A, NAD, lipoic acid, PreQ1, and heme metabolism, revealing a much broader capacity for cofactor production than previously assumed. We also found a CRISPR array lacking a cas‐motif. This orphan array may be linked to the identification of phage elements in our assembly, including two intact prophages and several remnants on the *Ca*Gg chromosome (Table S8), thereby explaining the remarkable enrichment of mobile genetic elements (MGEs) (Table 2). Remarkably, some of the MGEs are classified as plasmid‐origin gene families, and upon further examination, we identified several plasmid‐related genes in the *CaGg* genome, including some involved in plasmid replication, partitioning, stabilization, and conjugation, altogether suggesting possible distinct plasmid integrations into the *CaGg* genome (Table S9). None of these putative insertions result in coverage drops or changes in Hi‐C signals, indicating that these are not artifactual.

Overall, the MGEs cover 6.97% of the *CaGg* genome and are classified into five functional categories: Integration/Excision (IE, 102), Replication/Recombination/Repair (RRR, 21), Phage (P, 22), Stability/Transfer/Defense (STD, 47), and Transfer (T, 15). As observed in other Glomeromycotina endosymbionts (Sorwar *et al*., 2024), IE elements are enriched in the genome, suggesting that they are major contributors to genome plasticity in Glomeromycotina endosymbionts. The high mobilome content prompted us to analyze type II toxin‐antitoxin (TAs) modules, which are thought to stabilize MGEs (Chan *et al*., 2023) and mediate stress‐induced persistence in *Ca*Gg (Salvioli *et al*., 2017). The present assembly uncovered 41 new TAs (39 chromosomal and 8 on p*Ca*Gg01), up from 9 in earlier predictions (Salvioli *et al*., 2017) (Table S10).

## Discussion

In this work, we reported the first chromosome‐level assembly and 3D analysis of an AMF genome outside the Glomerales. This provided an unprecedented view into the biology of one of the largest and most repeat‐rich genomes known within the fungal kingdom and uncovered novel insights into the biology and evolution of its bacterial endosymbiont.

### A chromosomal view of a large and highly repetitive non‐model AMF

Combining long reads with chromatin capture methods has successfully helped assemble the large, highly repetitive *G. margarita* genome and its endosymbiont into a complete chromosome‐level assembly. This allowed us to demonstrate that AMF have more chromosomes than most other fungal relatives and that, with 43 chromosome‐level scaffolds, *G. margarita* likely has one of the highest reported chromosome counts to date in the fungal kingdom. Our chromosome annotation reveals that *G. margarita* encodes *c*. 30 211 genes and retains all the hallmarks of obligate biotrophy typical of AMF (Oliveira *et al*., 2024). These include a reduced set of cell wall‐degrading enzymes, the lack of fatty acid biosynthesis genes, the loss of thiamine synthesis genes, and the uptake of soluble sugars, such as glucose, which are essential compounds they obtain directly from their hosts (Tisserant *et al*., 2013; Morin *et al*., 2019). We also report a larger‐than‐expected set of proteins potentially involved in the dialogue with the host (Kloppholz *et al*., 2011; Voß *et al*., 2018; Yildirir *et al*., 2022; Teulet *et al*., 2023), suggesting an improved ability to establish symbioses with multiple plants, and we found primary evidence that *G. margarita* utilizes cobalamin produced by its endobacterial host, *Ca*Gg.

Beyond gene content, the chromosome‐level assembly provides a curated and comprehensive view of TE diversity in a genome long recognized for its repetitiveness and fragmentation (Venice *et al*., 2020). While previous work linked the large genome size of *G. margarita* primarily to LINE expansions (Venice *et al*., 2020), our refined TE annotation uncovers a substantial contribution of Class II elements, including DNA/TIR transposons and Mavericks in *G. margarita* genome biology. These DNA transposons have been implicated in gene duplication, genome restructuring, and regulatory innovation in fungi and other eukaryotes (Muszewska *et al*., 2019), suggesting that multiple TE classes, not only retrotransposons, continue to contribute to genome complexity in *G. margarita*. Our analyses also continue to support the view that, in addition to dictating genome evolution, the AMF TEs are major players in regulating gene expression during colonization, presumably due to their localization within regulatory regions and in proximity to candidate secreted proteins and effectors. It is noteworthy that different TE families are regulated in *G. margarita* and in *R. irregularis* strains during root colonization. This could mirror lineage‐specific regulatory adaptations in AMF and/or host plant‐driven TE regulations (Oliveira & Corradi, 2024). It will be important to determine whether the observed differences reflect direct effects of the endobacterium or indirect consequences of altered colonization dynamics within the tripartite interaction.

The genome analyses of a large AMF genome also reinforce the growing evidence that rDNA sequence diversity is significant within individual AMF genomes. This continues to highlight the challenges of using this locus alone for taxonomic resolution as divergent rDNA copies cluster by paralogy rather than by speciation and supports the need for population genetics‐based approaches in AMF taxonomy (Bruns *et al*., 2018; Corradi *et al*., 2025). Our evidence that some rDNA copies are likely inactive further exacerbates these challenges.

### Remarkable conservation in genome biology among AMF lineages

In addition to sharing losses in key genes associated with obligate biotrophy and high intragenomic rDNA divergence, the genome biology of *G. margarita* follows patterns conserved among distinct AMF lineages. All species investigated to date with Hi‐C have genomes partitioned into an A compartment with high gene density, expression, and repeat methylation, and a B compartment with low repeat methylation and an enrichment in secreted and effector proteins. This spatial organization of AMF chromatin likely maintains genome stability by silencing repeats in the A compartment, which is rich in housekeeping genes (Yildirir *et al*., 2022), while allowing the B compartment to serve as a hotspot for diversifying secreted proteins and effector‐encoding genes. Altogether, these features continue to underpin striking analogies in the genome biology and evolution of AMF and filamentous plant pathogens (Dong *et al*., 2015; Reinhardt *et al*., 2021). Despite the shared similarities, AMF genomes exhibit some notable epigenetic distinctions. For example, while the *R. irregularis* heterokaryons show a tripartite genome‐wide CpG methylation distribution (Sperschneider *et al*., 2023), the homokaryons and *G. margarita* display a bimodal methylation pattern (Yildirir *et al*., 2022; Manley *et al*., 2023). The reasons for these differences remain unknown, although it is tempting to speculate that the nuclear type (homokaryon vs heterokaryon) influences AMF methylation distribution patterns. Our work also identified notable distinctions between TADs in AMF and those of fungal relatives. For example, in the fungal species *Epichloë festucae*, AT‐rich, repeated blocks contribute to the formation of TADs (Winter *et al*., 2018), yet the repeat‐depletion at the boundaries we observed suggests an alternative mechanism for TAD establishment in AMF that is more consistent with reports from other eukaryotic lineages (Dixon *et al*., 2016; Li *et al*., 2022; Glavincheska & Lorrain, 2025; Kurbidaeva *et al*., 2025).

### A model that explains the co‐transcription of divergent rDNA operons in AMF

In most eukaryotes, rDNA genes exist as hundreds to thousands of identical copies scattered across different chromosomes, yet these copies are all transcribed in a spatially coordinated manner within the nucleolus (Grummt, 2003;Boisvert *et al*., 2007; Pederson, 2011). The localization of rDNA genes within the nucleolus has been primarily studied using microscopy techniques, such as fluorescence *in situ* hybridization (FISH) and immunofluorescence (Boisvert *et al*., 2007; Mancio‐Silva *et al*., 2010). Recently, 3C‐based methods, such as Hi‐C, have been employed to identify these interactions at a genome‐wide scale, providing an additional independent line of evidence for rDNA co‐localization (Rabuffo *et al*., 2024). In our work, the presence of fewer, divergent rDNA copies in *G. margarita* has likely facilitated their capture and separation by Hi‐C, thereby enabling the detection of their strong physical interactions, indicating that these genes co‐localize, presumably within the nucleolus, which may facilitate their co‐transcription and enhance ribosome biogenesis (Boisvert *et al*., 2007; Pederson, 2011). In addition, the identification of identical signals in *R. irregularis* (Yildirir *et al*., 2022) shows that the co‐localization of rDNA is a conserved mechanism of rDNA organization and coregulation in AMF.

### An improved view of endosymbiotic contribution to *G. margarita*

Our complete genome assembly and comparative analysis revealed that the *Ca*Gg genome comprises a large circular chromosome and two smaller complete circular plasmids, each with distinct ORI motifs, supported by HiFi and Hi‐C data and independent assemblers. As such, this work clarifies previous assumptions, which suggested that the *Ca*Gg genome is organized into 2 to 4 genomic units ranging between 1.4 and 2.5 Mb in size (Jargeat *et al*., 2004; Ghignone *et al*., 2012). Like other AMF endosymbionts (Bonfante & Desirò, 2017; Pawlowska *et al*., 2018; Uehling *et al*., 2023), the *Ca*Gg genome is smaller than that of free‐living *Burkholderia* relatives (Winsor *et al*., 2008), resulting in limited metabolic capabilities, including the inability to utilize glycolysis as an energy source and a limited capacity to biosynthesize essential amino acids, underscoring the AMF host's strong dependence.

The close‐knit relationship between *Ca*Gg and *G. margarita* is further emphasized by our finding that some fungal TEs are upregulated only in the presence of the endobacterium, and the identification of novel pathways through which *Ca*Gg may influence the host's fungal physiology. In particular, the presence of a complete heme biosynthesis pathway suggests an unsuspected role in supporting AMF host bioenergetics, consistent with observations in other host‐endosymbiont systems (Strübing *et al*., 2010). Indeed, heme is an essential cofactor for many proteins, including cytochromes of the mitochondrial electron transport chain, which are highly abundant in the *G. margarita* genomes. As such, we hypothesize that *Ca*Gg‐derived heme enhances the activity of respiratory complexes in the AMF host, potentially aligning with the higher ATP production and respiratory activity reported in endobacteria‐containing *G. margarita* compared to the cured line (Venice *et al*., 2017). In other filamentous fungi (Wang *et al*., 2024), heme also plays roles in growth, development, and stress adaptation, suggesting that *Ca*Gg‐encoded heme production could affect multiple aspects of AMF physiology beyond respiration. Future analyses will hopefully reveal how these novel endobacterial genes are regulated in response to environmental changes.

Another surprising discovery was that *Ca*Gg carries several MGEs, including prophages, expanding the viral pool within the *G. margarita* symbiotic system (Turina *et al*., 2018). While most bacteria rely on CRISPR‐Cas systems to defend against foreign DNA (Horvath & Barrangou, 2010; Rostøl & Marraffini, 2019), *Ca*Gg, like many endosymbionts, lacks a functional CRISPR‐Cas system (Burstein *et al*., 2016; Siozios *et al*., 2024). The presence of this orphan CRISPR array likely explains the high MGE content, including prophages, in the *Ca*Gg genome, and it indicates that CRISPR‐based defense was active in the Burkholderia‐related endobacteria ancestors before some lineages became obligate endosymbionts. Conversely, the enrichment of toxin‐antitoxin systems in *Ca*Gg might represent an alternative defense strategy in the absence of CRISPR‐Cas (Rostøl & Marraffini, 2019; Song & Wood, 2020).

### Conclusions

This study presents the first chromosome‐scale and 3D genome assembly of a non‐model AMF, revealing conserved principles of chromatin architecture among AMF despite dramatic differences in genome size and repeat content. Our findings confirm that A/B compartmentalization and TAD‐like structures are fundamental features of AMF genome organization, consistent across phylogenetically and ecologically distinct AMF species (Horsch *et al*., 2023), supporting coordinated gene expression and genome stability. It will now be important to assess the dynamics of these compartments and TAD‐like structures across AMF life stages or under varying environmental conditions. We also uncovered a novel mechanism for rDNA co‐localization within the nucleolus, which likely facilitates ribosome biogenesis despite extensive rDNA sequence divergence – a hallmark of AMF genomes.

Beyond fungal chromatin biology, our work sheds light on new functional contributions of the obligate endobacterium *Ca*Gg by revealing a multipartite architecture enriched in mobile genetic elements, underscoring its genomic plasticity that cannot be offset by an incomplete CRISPR‐Cas system. Additionally, we identify novel pathways for essential cofactors, including heme and cobalamin, suggesting a potential role in enhancing host bioenergetics and stress resilience, a hypothesis that would be interesting to test experimentally. The observation that *Ca*Gg presence modulates transposable element activity in *G. margarita* further highlights a complex interplay between host and symbiont at the epigenetic and genome stability levels. As multiple AMF species carry bacterial endosymbionts (Oliveira *et al*., 2025), future studies should aim to acquire similarly complete genomes and thus fully uncover the diversity of intricate biological and biochemical mechanisms that exist between these partners and their plant hosts.

Together, these findings point to a model in which chromatin architecture, rDNA organization, and endosymbiont‐derived metabolic capabilities collectively shape the biology and ecological success of AMF. Future work should explore how these interactions respond to environmental cues and influence plant fitness, including in early‐branching lineages, to provide complete insights into the molecular foundations of one of the most impactful plant symbioses.

## Competing interests

None declared.

## Author contributions

KM: design of the research; performance of the research; data analysis, interpretation; and writing the manuscript. JO: design of the research; performance of the research; data analysis; interpretation; and writing the manuscript. FM: data analysis; interpretation; feedback on the manuscript. AS: collection of fungal material. MN: collection of fungal material. LL: feedback on the manuscript. SG: interpretation; feedback on the manuscript. GY: data analysis; interpretation. YW: data analysis; interpretation. PB: collection of fungal material; interpretation; and feedback on the manuscript. NC: design of the research and interpretation; and writing the original manuscript, funding acquisition.

## Disclaimer

The New Phytologist Foundation remains neutral with regard to jurisdictional claims in maps and in any institutional affiliations.

## Supporting information


**Fig. S1** Placement of *Gigaspora margarita* within the fungal phylogeny.
**Fig. S2** rDNA phylogeny of *Gigaspora* species.
**Fig. S3** Genome‐wide Hi‐C contact maps of *Gigaspora margarita* and *Rhizophagus irregularis*.
**Fig. S4** Hi‐C contact maps showing A/B compartments in *Gigaspora margarita* chromosomes at a 50 kb resolution in −*Ca*Gg and +*Ca*Gg germinating spores.
**Fig. S5** Boxplots showing variation of expression across conditions: ERM, germinating spores, and *in planta* (lotus) within TAD and non‐TAD regions.
**Fig. S6** Hi‐C contact maps of *Candidatus* Glomerobacter gigasporarum (*Ca*Gg) chromosome.

**Table S1** Hi‐C library summary statistics in *Gigaspora margarita* for −*Ca*Gg and +*Ca*Gg samples.
**Table S2** Secreted proteins and effectors in chromosome‐level assemblies of *Gigaspora margarita* and *Rhizophagus irregularis*.
**Table S3** Missing Glomeromycota Core Genes (MGCGs).
**Table S4** Carbohydrate‐active enzymes (CAZymes) families.
**Table S5** B12‐dependent enzymes in *Gigaspora margarita* identified using homologs from *Rhizophagus irregularis*.
**Table S6** Location and methylation levels of rDNA units in the *Gigaspora margarita* genome.
**Table S7** Distribution of secreted proteins and effector genes in A/B compartments of *Gigaspora margarita*.
**Table S8** Putative prophage regions in *Ca*Gg.
**Table S9** Putative plasmid genes in the *Ca*Gg chromosome.
**Table S10** Putative type II toxin‐antitoxin modules in *Ca*Gg.
Please note: Wiley is not responsible for the content or functionality of any Supporting Information supplied by the authors. Any queries (other than missing material) should be directed to the *New Phytologist* Central Office.

## Data Availability

The sequencing data used in our study are available in GenBank under BioProject PRJNA1364746. The chromosome assemblies and annotations are available at Zenodo: https://doi.org/10.5281/zenodo.18236849. All the scripts used can be accessed here: https://github.com/kenmurithi/Mugambi‐2026‐AMF‐endosymbiont‐genomics.
