# Supplementary material for: The 3D genome of Gigaspora margarita unveils stable chromatin and nucleolar organization and symbiont‐dependent genome dynamics
Source: New Phytol. 2026 Mar 20;251(5):2799–817. doi: 10.1111/nph.71100 (PMC13443276; doi:10.1111/nph.71100)
Supplement: Supplementary file 1 [file NPH-251-2799-s002.pdf]

New Phytologist **Supporting Information**

Article title: The 3D Genome of *Gigaspora margarita* Unveils Stable Chromatin and Nucleolar Organization and Symbiont-Dependent Genome Dynamics

Authors: Ken Mugambi, Jordana Oliveira, Franco Magurno, Alessandra Salvioli di Fossalunga, Mara Novero, Luisa Lanfranco, Stefano Ghignone, Gökalp Yildirir, Yan Wang, Paola Bonfante, Nicolas Corradi

Article acceptance date: 25 February 2026

The following Supporting Information is available for this article:

Fig. S1. Placement of *G. margarita* within the fungal phylogeny.

Fig. S2. rDNA phylogeny of *Gigaspora* species

Fig. S3. Genome-wide Hi-C contact maps of *G. margarita* and *R. irregularis*.

Fig. S4. Hi-C contact maps showing A/B compartments in *G. margarita* chromosomes at a 50 kb resolution in -*Ca*Gg and + *Ca*Gg germinating spores.

Fig. S5. Boxplots showing variation of expression across conditions: ERM, germinating spores, and *in planta* (lotus) within TAD and non-TAD regions.

Fig. S6. Hi-C contact maps of *Candidatus* Glomerobacter gigasporarum (*Ca*Gg) chromosome.

Table S1. Table S1: Hi-C library summary statistics in *G. margarita* for -*Ca*Gg and +*Ca*Gg samples.

Table S2. Secreted proteins and effectors in chromosome-level assemblies of *G. margarita* and *R. irregularis*.

Table S3. Missing Glomeromycota Core Genes (MGCGs).

Table S4. Carbohydrate-active enzymes (CAZymes) families.

Table S5. B12-dependent enzymes in *G. margarita* identified using homologs from *R. irregularis.*

Table S6. Location and methylation levels of rDNA units in the *G. margarita* genome.

Table S7. Distribution of secreted proteins and effector genes in A/B compartments of *G. margarita.*

Table S8. Putative prophage regions in *Ca*Gg.

Table S9. Putative plasmid genes in the *Ca*Gg chromosome.

Table S10. Putative Type II toxin-antitoxin modules in *Ca*Gg.

Figure S1. Placement of *G. margarita* within the fungal phylogeny. Maximum likelihood phylogenetic tree showing the placement of *Gigaspora margarita* on the fungal tree of life based on 702 single-copy orthologs. Bootstrap support values are indicated on each branch.

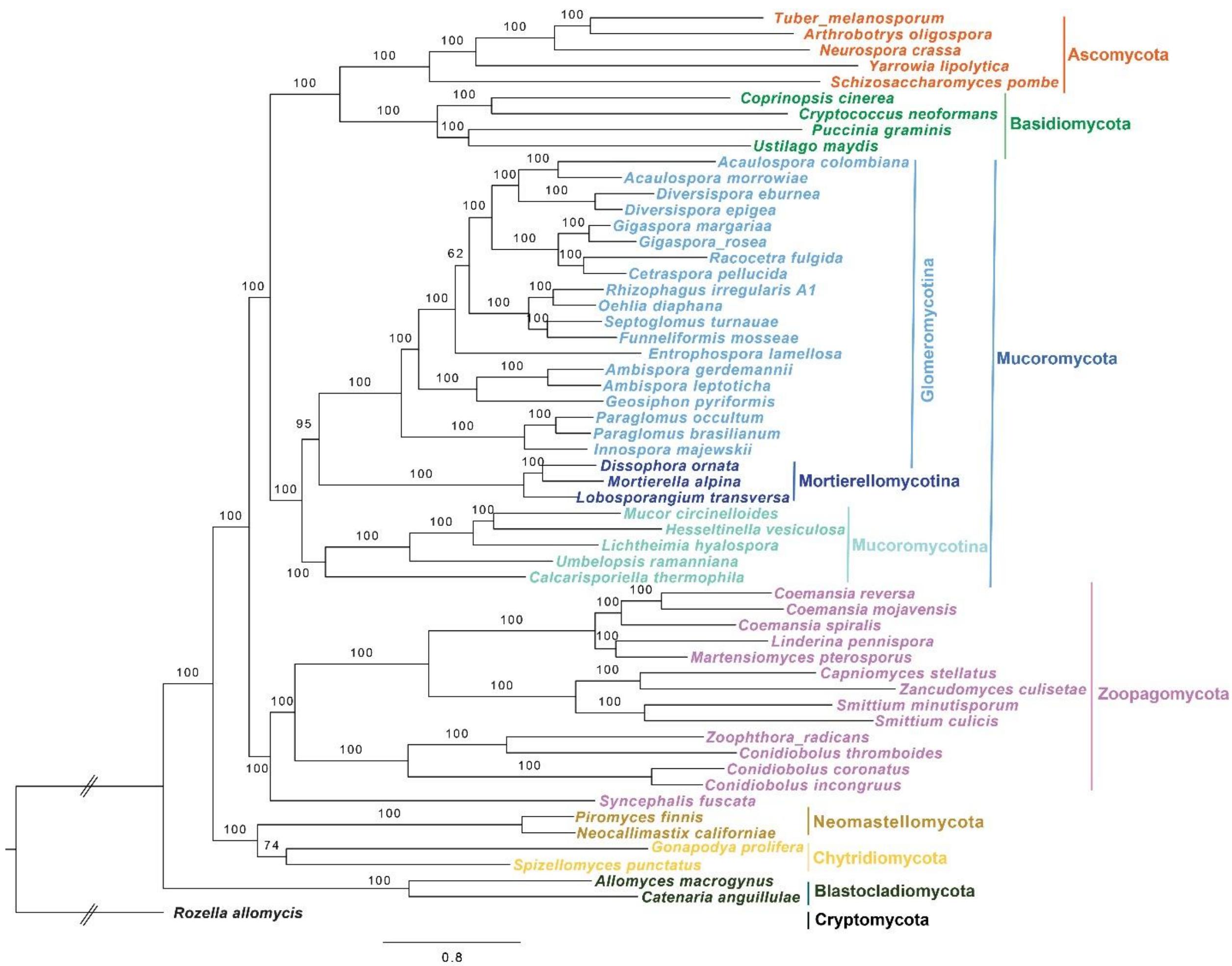

Figure S2. rDNA phylogeny of *Gigaspora* species. Phylogram generated from Maximum Likelihood (ML) analyses showing the phylogenetic relationships among *G. margarita* and other *Gigaspora* species possessing rDNA sequences. Sequences of *Dentiscutata savannicola* and *Fuscutata heterogama* were used to root the tree. Different colours for the external branches indicate the species shown in the legend. Internal branches are coloured to match the same species notation as their descendant leaves when those leaves are of a single species. Bootstrap values, ultrafast bootstrap values, and posterior probabilities ≥ 90, 90, and 0.98, respectively, are indicated above or below the branches. The bar represents 0.02 expected changes per site per branch.

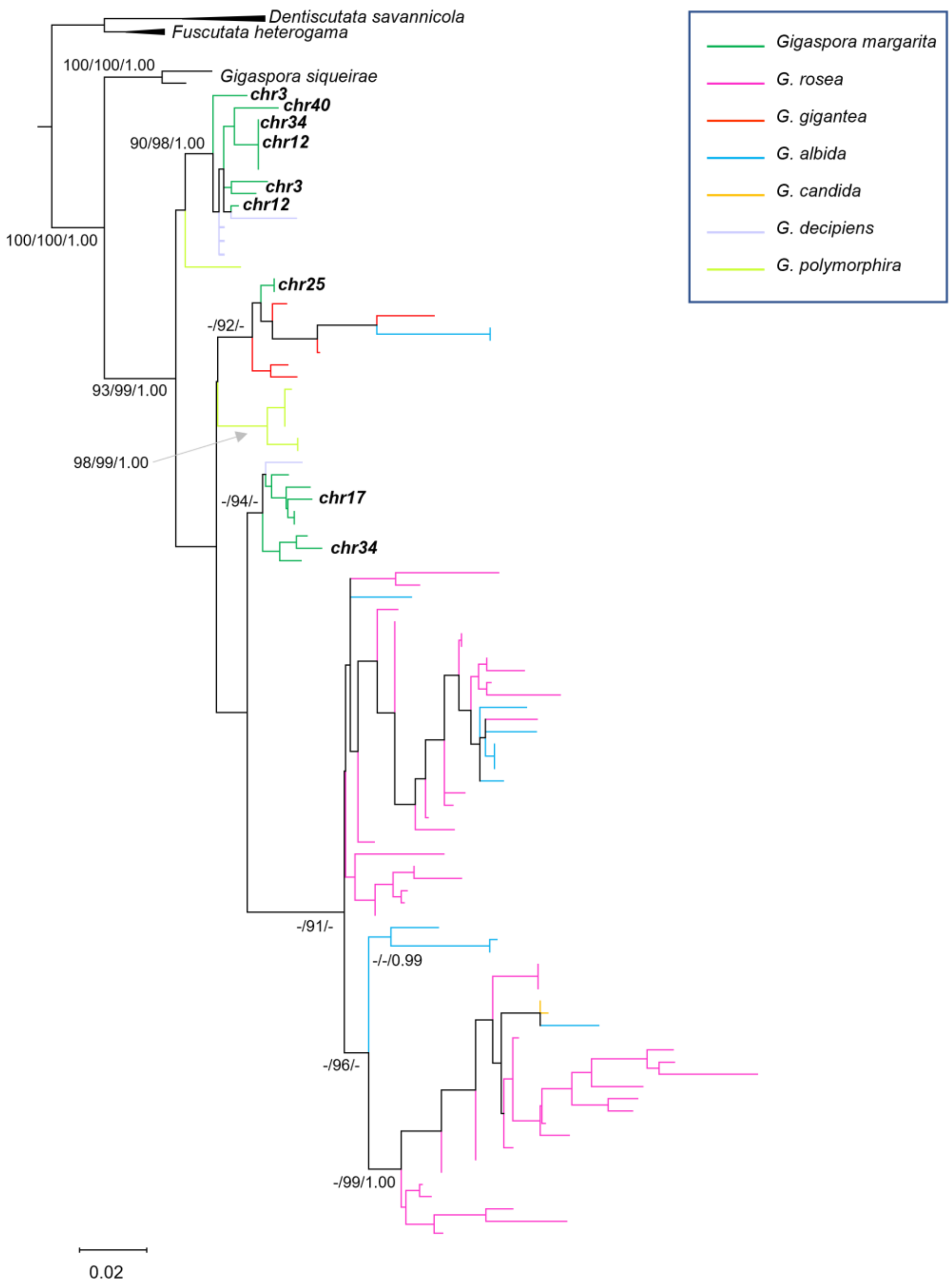

Figure S3. Genome-wide Hi-C contact maps of *G. margarita* and *R. irregularis*. The black squares represent chromosomes sorted by size. The colour intensity corresponds to interaction frequencies between loci, with darker red indicating high contact probability and white signifying low or no interactions. The circles highlight physical interactions between rDNA across different chromosomes.

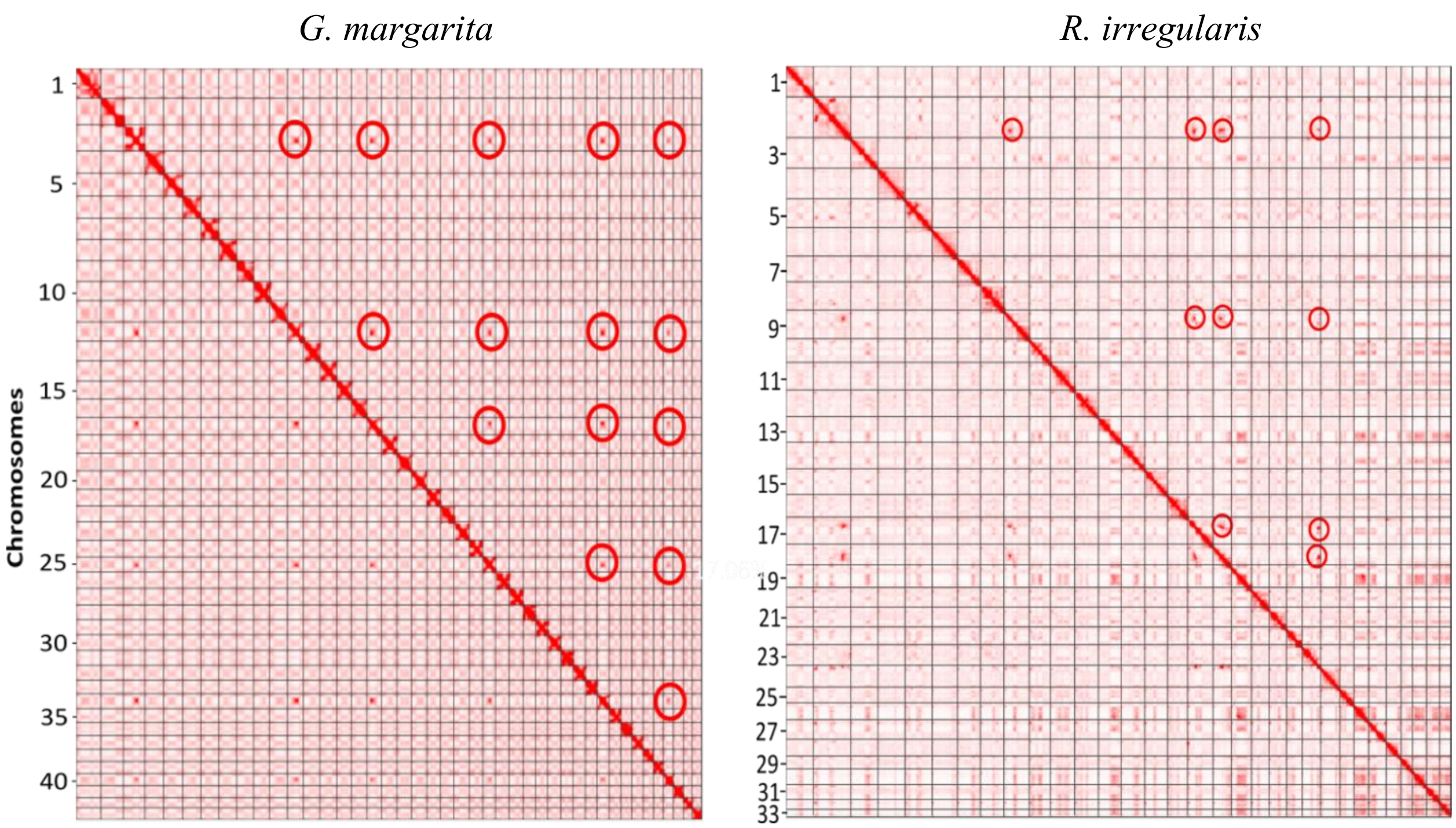

Figure S4. Hi-C contact maps showing A/B compartments in *G. margarita* chromosomes at a 50 kb resolution in -*Ca*Gg and +*Ca*Gg germinating spores. Regions that interact more frequently are visualized as brighter squares on the contact map.

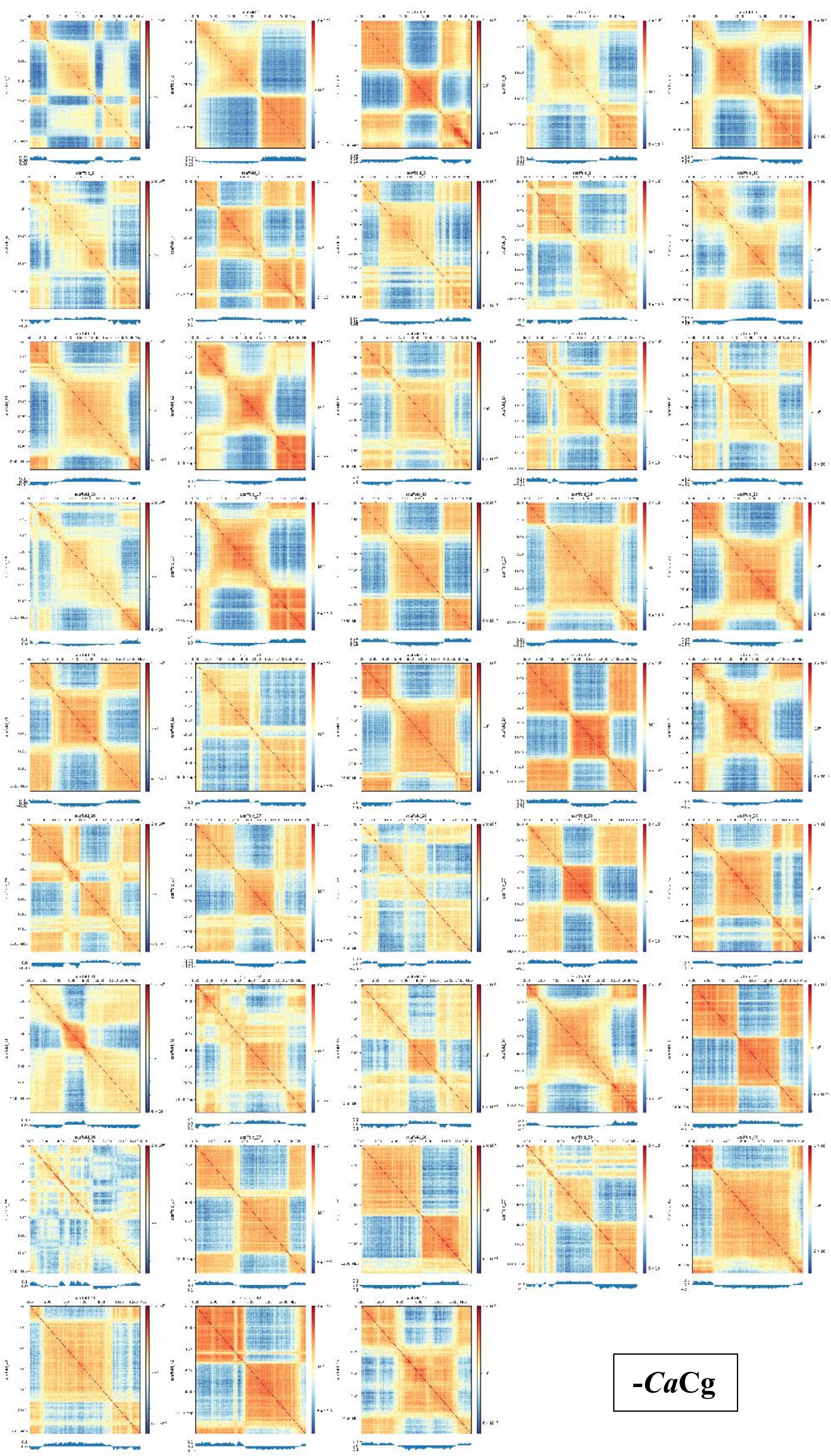

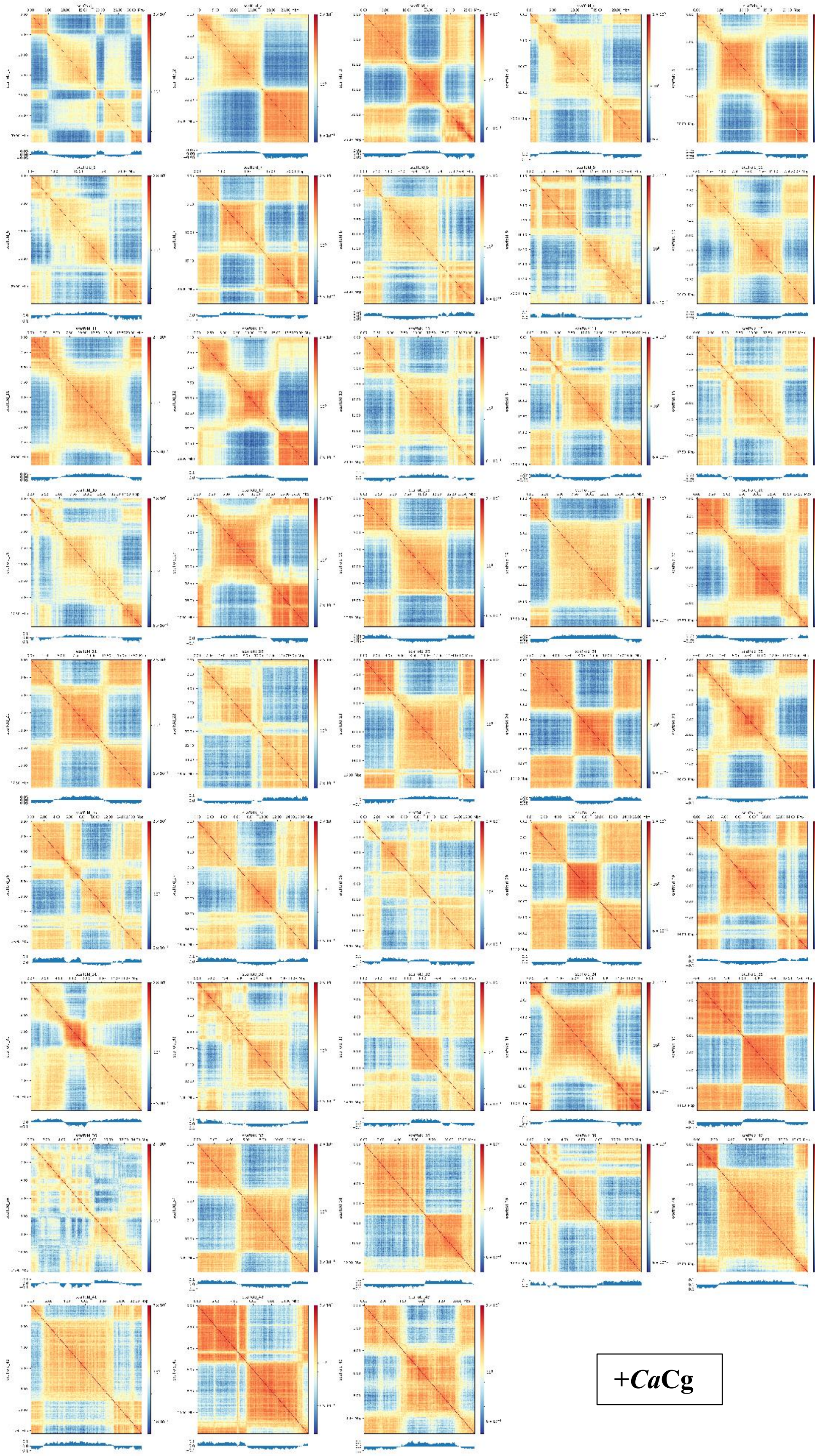

+*Ca*Cg

Figure S5. Boxplots showing variation of expression across conditions: ERM, germinating spores, and *in planta* (lotus) within TAD and non-TAD regions. Boxes show the first quartile (25%), the middle black line (50%), and the third quartile (75%). The whiskers extend to 1.5× the box length, and the outliers are represented as dots. Asterisks above the boxplots indicate significant differences between the A/B compartments ($p < 0.05$, Wilcoxon rank-sum test).

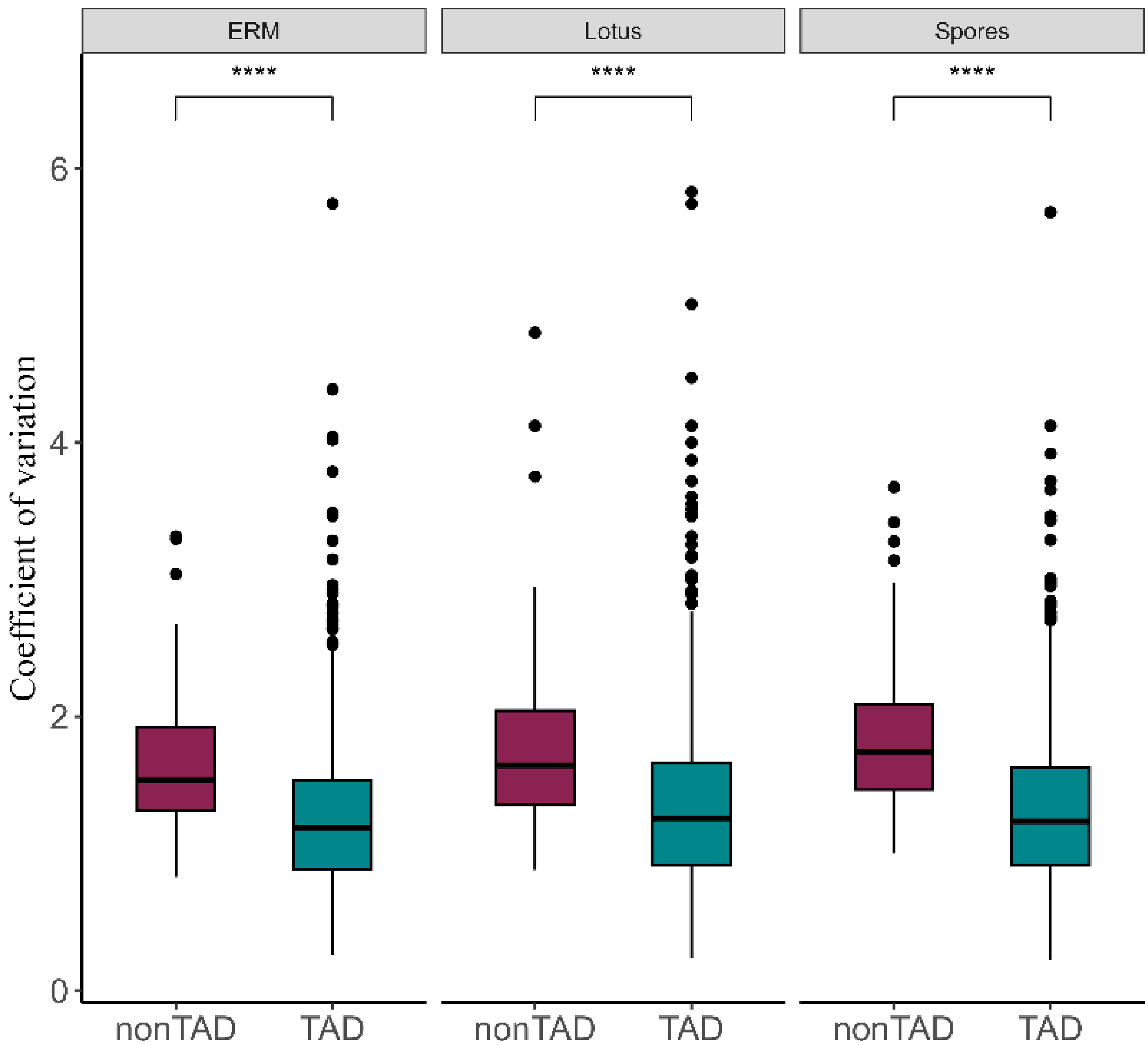

Figure S6. Hi-C contact maps of *Candidatus* Glomerobacter gigasporarum (*Ca*Gg) chromosome. Regions that interact more frequently are visualized as brighter squares on the contact map

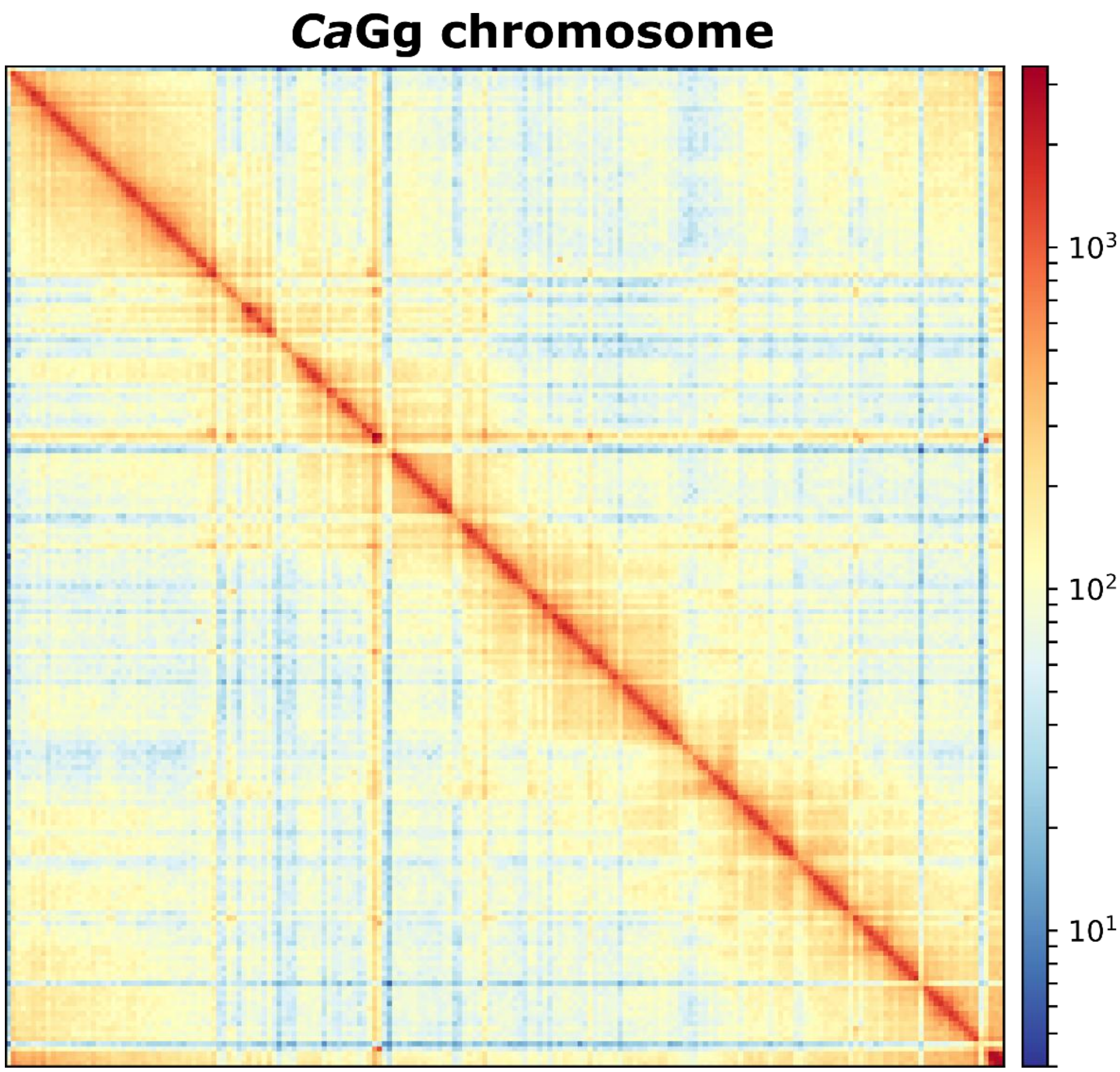

Table S1. Table S1: Hi-C library summary statistics in *G. margarita* for -*Ca*Gg and +*Ca*Gg samples.

See the accompanying excel spreadsheet

Table S2. Secreted proteins and effectors in chromosome-level assemblies of *G. margarita* and *R. irregularis*.

See the accompanying excel spreadsheet

Table S3. Missing Glomeromycota Core Genes (MGCGs).

See the accompanying excel spreadsheet

Table S4. Carbohydrate-active enzymes (CAZymes) families.

See the accompanying excel spreadsheet

Table S5. B12-dependent enzymes in *G. margarita* identified using homologs from *R. irregularis.*

See the accompanying excel spreadsheet

Table S6. Location and methylation levels of rDNA units in the *G. margarita* genome.

See the accompanying excel spreadsheet

Table S7. Distribution of secreted proteins and effector genes in A/B compartments of *G. margarita.*

See the accompanying excel spreadsheet

Table S8. Putative prophage regions in *Ca*Gg.

See the accompanying excel spreadsheet

Table S9. Putative plasmid genes in the *Ca*Gg chromosome.

See the accompanying excel spreadsheet

Table S10. Putative Type II toxin-antitoxin modules in *Ca*Gg.

See the accompanying excel spreadsheet
